# Supplementary material for: Functional and regulatory diversification of Period genes responsible for circadian rhythm in vertebrates
Source: G3 (Bethesda). 2024 Jul 19;14(10):jkae162. doi: 10.1093/g3journal/jkae162 (PMC11457068; doi:10.1093/g3journal/jkae162)
Supplement: jkae162_Supplementary_Data [file jkae162_supplementary_data.zip › Supplemental_Figures_and_Legends_G3-2024-405148.docx]

**Supplementary Materials**

**Table S1. Master table of investigated species.**

**Table S2. Excluded species because of “N” in the *Period* genes.**

**Table S3. Variables in continental species.**

**Table S4. Variables in marine species.**

**Table S5. Coefficient of variation of gene numbers in gene families**

**​​Table S6. MEME selection detection results for *Period* genes.**

**Table S7. The length of each *Period* gene**

**Table S8. NCBI Conserved Domain search results**

**Table S9. MEME motif discovery results**

**Figure S1 RELAX result of salmonid *per3* gene.**

**Figure S2 The distribution of coefficient of gene copy number variation of each gene family across taxa**

**Figure S3 Brain *PER* gene expression in humans.**

**Figure S4 Diversifying selection signature in Period genes in vertebrates.**

**File S1. An R script for gene sequence curation and visualization and fetching upstream sequences**

**File S2. A Timetree-based species tree.**

**File S3. *Per1* species-gene reconciled tree**

**File S4. *Per2* species-gene reconciled tree**

**File S5. *Per3* species-gene reconciled tree**

**File S6. The phylogenetic tree of PER proteins.**

Supplementary Figures

**
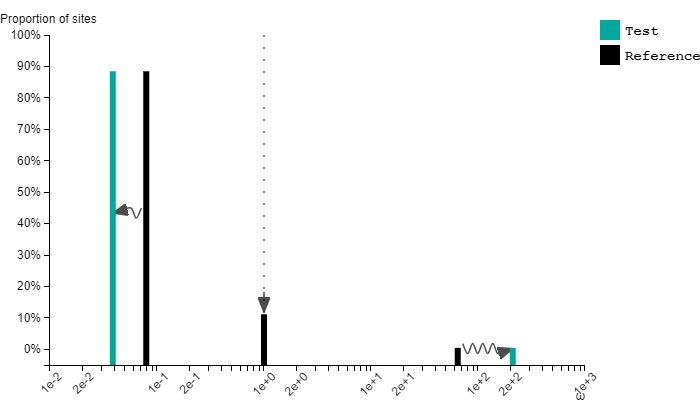
**

**Figure S1:** Signature of relaxed selection on per3 sequence in salmon.
Each bar represents the proportion of sites (y-axis) corresponding to the ω values (x-axis) indicated for both the test (green) and reference (black) groups. The arrows indicate the changes in ω values estimated by the model of the software. Figure retrieved from Datamonkey (https://www.datamonkey.org/).


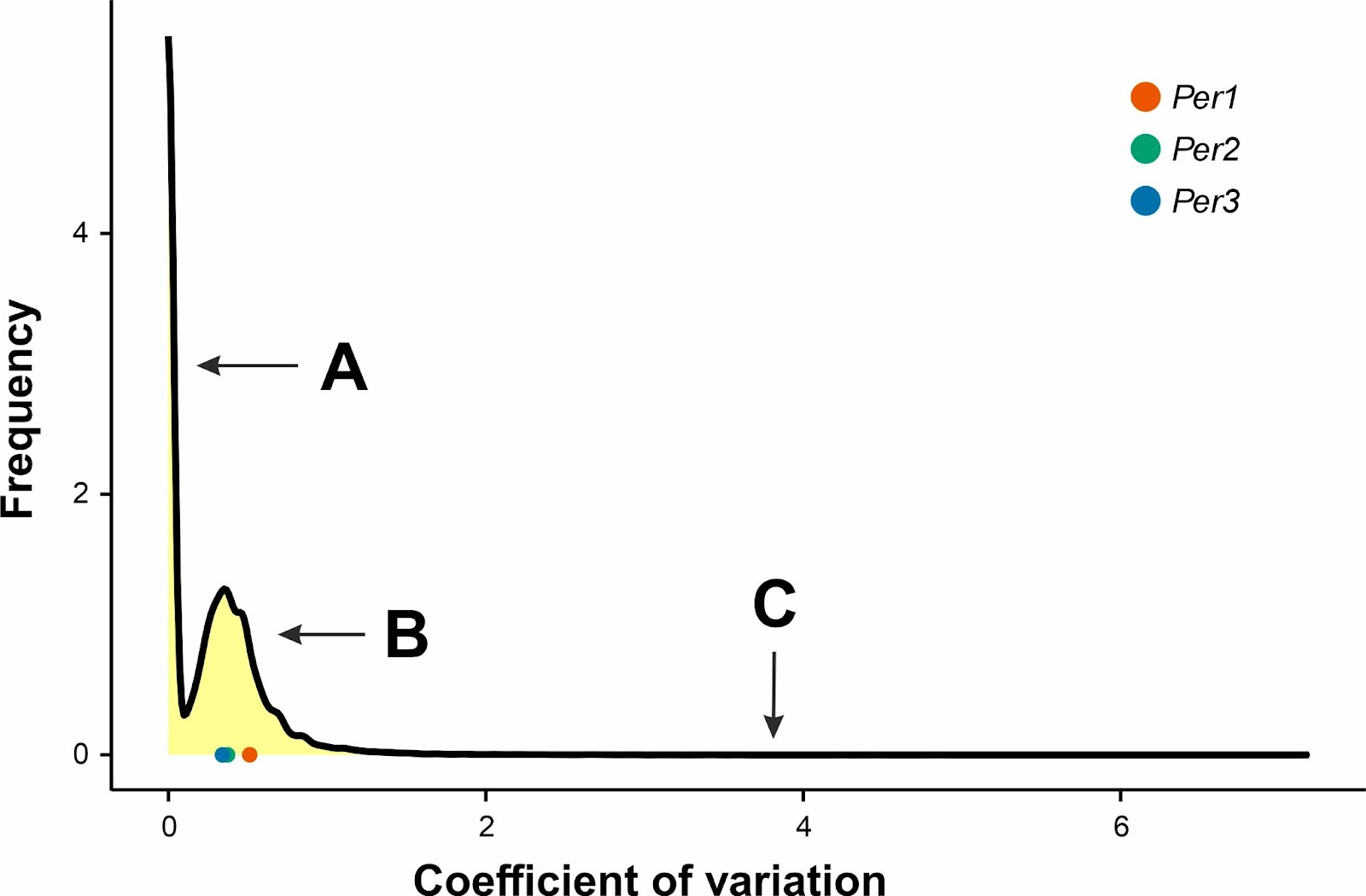


**Figure S2:** The distribution of coefficient of gene copy number variation of each gene family across taxa. We found three gene groups with very low variation (A), medium variation (B) and high variation (C). per genes fell in the medium variation cluster

**Figure S3** Brain *PER* gene expression during human development, retrieved from the data on Brainspan (https://www.brainspan.org/).


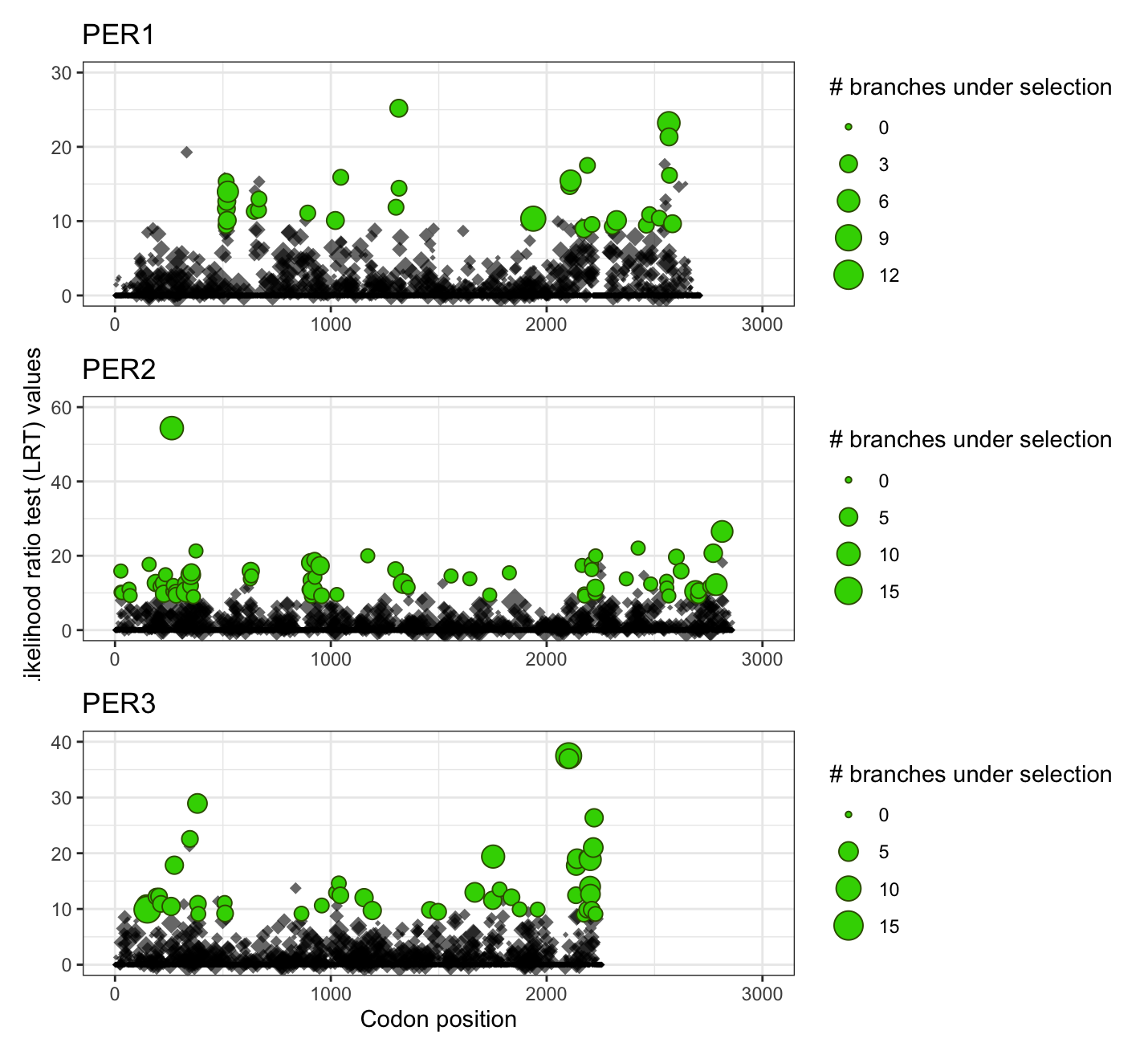


**FigureS4.** Diversifying selection signature in Period genes in vertebrates detected by MEME (Mixed Effects Model of Evolution) (77) on Datamonkey version 2.0 (78)

Codons with p-value < 0.01 and branches under selection > 1 are highlighted with green dots.
